# Experimental Design-Based Functional Mining and Characterization of High-Throughput Sequencing Data in the Sequence Read Archive

**DOI:** 10.1371/journal.pone.0077910

**Published:** 2013-10-22

**Authors:** Takeru Nakazato, Tazro Ohta, Hidemasa Bono

**Affiliations:** Database Center for Life Science (DBCLS), Research Organization of Information and Systems (ROIS), Tokyo, Japan; Cairo University, Egypt

## Abstract

High-throughput sequencing technology, also called next-generation sequencing (NGS), has the potential to revolutionize the whole process of genome sequencing, transcriptomics, and epigenetics. Sequencing data is captured in a public primary data archive, the Sequence Read Archive (SRA). As of January 2013, data from more than 14,000 projects have been submitted to SRA, which is double that of the previous year. Researchers can download raw sequence data from SRA website to perform further analyses and to compare with their own data. However, it is extremely difficult to search entries and download raw sequences of interests with SRA because the data structure is complicated, and experimental conditions along with raw sequences are partly described in natural language. Additionally, some sequences are of inconsistent quality because anyone can submit sequencing data to SRA with no quality check. Therefore, as a criterion of data quality, we focused on SRA entries that were cited in journal articles. We extracted SRA IDs and PubMed IDs (PMIDs) from SRA and full-text versions of journal articles and retrieved 2748 SRA ID-PMID pairs. We constructed a publication list referring to SRA entries. Since, one of the main themes of -omics analyses is clarification of disease mechanisms, we also characterized SRA entries by disease keywords, according to the Medical Subject Headings (MeSH) extracted from articles assigned to each SRA entry. We obtained 989 SRA ID-MeSH disease term pairs, and constructed a disease list referring to SRA data. We previously developed feature profiles of diseases in a system called “Gendoo”. We generated hyperlinks between diseases extracted from SRA and the feature profiles of it. The developed project, publication and disease lists resulting from this study are available at our web service, called “DBCLS SRA” (http://sra.dbcls.jp/). This service will improve accessibility to high-quality data from SRA.

## Introduction

High-throughput sequencing technology is a powerful technique for determination of an entire genome sequence and quantification of the transcriptome at base-pair resolution with a large dynamic range. The sequencers using massively parallel sequencing technology, also called next-generation sequencer (NGS), drastically reduce the cost and time of sequencing, compared with previous methods, and is rapidly becoming the technology of choice for such purposes [1]. This type sequencers yields a vast quantity of captured images, in-process files, and numerous sequence reads, requiring an extensive amount of disk space [2]. However, such data are important for researchers and should be shared, as are the nucleotide sequences in GenBank and microarray data in the Gene Expression Omnibus (GEO). Thus, raw sequencing data from a high-throughput sequencing platform are submitted into a public international archive resource called the Sequence Read Archive (SRA) as a primary data archive. The SRA database is maintained by the National Center for Biotechnology Information (NCBI), the European Bioinformatics Institute (EBI), and the DNA Data Bank of Japan (DDBJ) as NCBI SRA [3], the European Nucleotide Archive (ENA) [4], and DDBJ SRA (DRA) [5], respectively. The archived data is collaboratively synchronized by these three institutes as part of the International Nucleotide Sequence Database Collaboration (INSDC) [6], thus researchers can search and download high-throughput sequencing data submitted to one of the institutes from the website of others. As of January 2013, data from more than 14,000 projects have been archived in SRA. SRA accepts the data produced by various sequencing platforms such as Illumina HiSeq 2000, Illumina MiSeq, Illumina Genome Analyzer IIx, Roche 454 GS FLX, Applied Biosystems SOLiD 5500xl, Complete Genomics, Ion Torrent PGM, and PacBio RS.

The archived SRA data contains not only raw read sequences but also information on the experimental design including project titles, species or cell lines, names of samples, and sequencing platforms as metadata. The metadata consists of six files in XML format: submission, study, experiment, run, sample, and analysis (Figure S1, http://trace.ddbj.nig.ac.jp/dra/metadata_e.shtml). For example, the title and abstract of a project is described in the "study" file, the experimental conditions and sequencing platforms are included in the "experiment" file, and scientific names and sample preparation methods are recorded in the "sample" file. Researchers can search SRA database, download data to compare with their own, and analyze archived data using their own software. However, the structure of SRA data is complex, and each submission has not all of six types of metadata files. Since additional experiments assigned to a previous project are often submitted as a new submission, and raw sequence data under a same project is divided to multiple directories on the FTP site. Furthermore, resultant data is usually giga-byte order, thus downloading sequence data from SRA is time-consuming. Further, the data qualities depend on their sequencing platforms and occasionally of questionable quality because anyone can submit data to SRA with no quality check. To overcome these difficulties, we constructed ID mapping tables and a list of publications that refer to high-throughput sequencing data. By means of this list, researchers can select captured sequencing data of high quality.

A major aim of -omics analyses is to understand disease mechanisms. In SRA, a large quantity of data relevant to diseases is archived. Therefore, we characterized and indexed SRA entries by disease keyword, using the Medical Subject Headings (MeSH), which is the National Library of Medicine’s (NLM’s) controlled vocabulary for indexing articles [7]. We provide the constructed lists on a web service, allowing researchers to easily find disease-relevant SRA entries.

## Methods

### Indexing each submission to a corresponding project in SRA database

**Figure 1:**
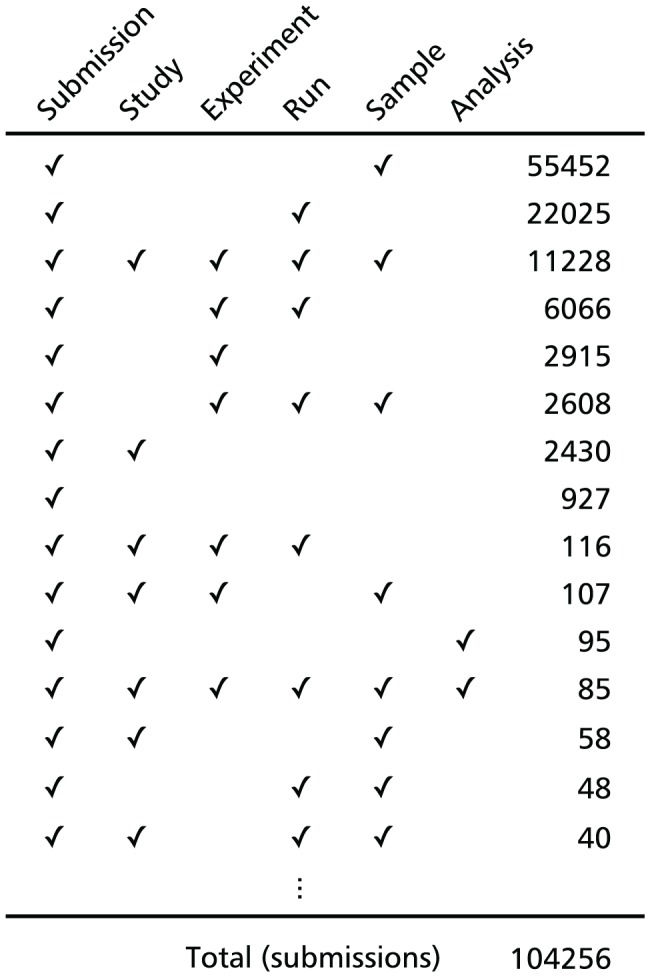
The presence or absence of six objects of Sequence Read Archive (SRA) metadata for each submission (top 15). The experimental designs including project titles, sequencing platforms and sample species are archived in SRA along with raw sequence data as six types of XML files: submission, study, experiment, run, sample and analysis. Analysis files are optional for submission. Each submission has not all those objects of metadata.

We downloaded all of the metadata in the SRA database from the DDBJ FTP site (ftp://ftp.ddbj.nig.ac.jp/ddbj_database/dra/) in March 2012. The experimental information including sequencing platforms and species of samples are separately recorded in six types of XML files: submission, study, experiment, run, sample and analysis (Figure S1). However, in the SRA database, each submission has not all those objects of metadata (Figure 1). The relationships between objects are often one-to-many, and raw sequence data under a same project is archived to separate directories. Since the corresponding IDs of other types of objects are described in each file as a reference link, we extracted each type of SRA ID (*e*.*g*., SRP000001 for “study”, SRX000001 for “experiment”, and SRS000001 for “samples”) from the metadata XML files and determined the connections among corresponding IDs. Accordingly, we constructed an ID mapping table.

### Articles extraction related to each SRA entry

We constructed a publication list that refers SRA data to assist to access high-throughput sequencing data of sufficiently high quality for analysis. A schematic view of the pipeline for generating SRA-PMID pairs is shown in Figure S2. We first retrieved the PubMed IDs (PMIDs) cited in the reference sections of the entire downloaded SRA metadata. Next, we extracted SRA IDs from journal articles in MEDLINE. Many IDs of external databases, including GenBank and OMIM, referred to in journal articles are cited in the external database section of MEDLINE, but the SRA IDs are not contained in this section. Therefore, we extracted SRA IDs from the full-text versions of articles in PubMed Central (PMC) and the websites of the journals that were freely available for parsing using regular expression pattern matching. In particular, we focused on articles assigned with MeSH term of “High-throughput Nucleotide Sequencing”. The SRA IDs extracted from journal articles are often not the same IDs used for submissions (*i*.*e*., start from SRA, ERA and DRA) or study (*i*.*e*. SRP, ERP and DRP), but are the IDs used for experiment (start from SRX, ERX and DRX) or run (SRR, ERR and DRR). Thus, we converted the extracted IDs to the corresponding SRA study IDs by the ID mapping table previously constructed.

Some transcriptome data captured by massively parallel sequencers are submitted not only to SRA but also to the GEO, and reference articles often cite GEO IDs as links to the captured data. Therefore, in addition to SRA IDs, we retrieved the GEO IDs and their corresponding referring PMIDs, using the same methods as described above, from the entire set of GEO data downloaded from the NCBI FTP site (ftp://ftp.ncbi.nlm.nih.gov/pub/geo/), and PMC article data. Referred GEO IDs contain a GEO dataset start from GDS, and the GEO series has a prefix of GSE. To convert the GEO IDs to the corresponding SRA IDs, we extracted SRA IDs from the GEO data and GEO IDs from the SRA metadata, and then constructed pairs of SRA and GEO IDs. Accordingly, we constructed a publication list referring to SRA data, showing publication title, journal name, PMID and referring SRA ID and data title.

### Characterization of each SRA entry by disease keywords

To characterize each SRA entry by other criteria, we used the MeSH controlled vocabulary. MeSH contains more than 23,000 keywords and hierarchically categorized into 15 concepts including “disease”, “chemicals and drugs”, and “anatomy”. It is originally curated for indexing MEDLINE articles by NLM.

We obtained MeSH terms (2012 release) from the NLM website (http://www.nlm.nih.gov/mesh/meshhome.html). We extracted assigned MeSH terms from retrieved journal articles, and then constructed pairs of SRA IDs and MeSH terms. We divided these SRA-MeSH pairs according to MeSH categories, such as diseases and chemicals, and obtained disease-relevant SRA entries by restricting these pairs to MeSH terms belonging to the "Disease" [C] branch or "Mental Disorders" [F03] sub-branch. To visualize the associations between SRA entries and relevant MeSH terms, we developed an association list showing the SRA IDs, SRA titles, relevant diseases and PMIDs from the source articles. We also generated a frequency list, according to the number of SRA entries associated with each disease, which allows researchers to restrict the list to a sub-category of disease, including neoplasms and mental disorders.

We previously constructed feature profiles of diseases referred to in Online Mendelian Inheritance in Man (OMIM) entries [8] by extracting disease MeSH terms from journal articles related to each OMIM entry. The feature profiles are available at our web service, which is called the Gene and Disease Features Ontology-based Overview System (Gendoo) (http://gendoo.dbcls.jp/) [9], [10]. We generated hyperlinks to corresponding Gendoo entries for each disease in the generated SRA-disease list by converting MeSH terms to their corresponding OMIM entries with Disease Ontology (DO) [11]; these were released in September 2012.

Diseases are often referred to by lexical variations, such as "lung cancer" and "lung neoplasms". To cope with this multiplicity of terms, we constructed a hierarchical tree view of MeSH disease terms so that researchers can search for SRA entries associated with a particular disease by referring to the MeSH hierarchy.

### Website implementation

We developed the web service called DBCLS SRA to make public high-throughput sequencing data more searchable and usable. DBCLS SRA provides a project list categorized by study types, sequencing platforms, and sample species. Additionally, it offers a publication list and a disease list.

The SRA data at INSDC databases is growing so fast, thus DBCLS SRA data updated weekly.

DBCLS SRA can be openly accessed at http://sra.dbcls.jp/ under Creative Commons Attribution 2.1 Japan license (http://creativecommons.org/licences/by/2.1/jp/deed.en).

## Results

### Indexing each submission to a corresponding project in SRA database

The SRA database is a primary archive of public high-throughput sequencing data, and provides experimental designs such as project titles and sequencers along with raw sequences as six objects of metadata XML files. As of January 2013, there have been more than 85,000 submissions in SRA database, comprising approximately 14,000 projects. In SRA, additional experiments are often assigned to a previous project and deposited as a new submission, thus the number of submission exceeds that of projects, and many submissions contain only a partial set of metadata files, even excluded “analysis” files from consideration, which are optional for submission (Figure 1). Therefore, we established connections between each type of metadata and constructed an ID mapping table. We provide a project list according to this table on our website, the DBCLS SRA. Researchers can sort and narrow the list by project types (*e*.*g*., whole genome sequencing and transcriptome analysis), species, and sequencing platforms, and can sort the data by scale and submission date. Table S1 shows the project list sorted according to the number of experiment files in a project.

### Extracting articles and constructing a publication list

The captured raw sequences in SRA are not always of a quality sufficient for meaningful analysis. Thus, we assessed the importance of each SRA entry according to the presence or absence of journal articles referring to it. To construct this publication list, we first obtained reference PMIDs from metadata XML files of SRA, and retrieved the 2666 pairs of SRA IDs and PMIDs. Then we extracted SRA IDs from full texts of journal articles in PMC and on the websites of the journals, and retrieved 863 SRA ID-PMID pairs (0.88 precision and 0.97 recall). The SRA IDs extracted from journal articles are often not the IDs used for submissions or study, thus we converted the extracted IDs to their corresponding study IDs using the ID mapping table previously constructed. The pairs of false positive were omitted from a publication list because there is no corresponding project information including SRA study ID, study title and sequencing platform name. Although we used regular expression pattern matching to retrieve SRA IDs from PMC articles, many methods are reported for extracting IDs of biological databases such as GEO and PDB [12]–[14]. We will attempt to apply these methods to extract SRA IDs from PMC. Even though we attempted to extract SRA IDs from journal articles in PMC, IDs are frequently described in supplementary files, which is located on journal’s web site as PDF format, so that we cannot obtain such IDs. In addition, the full text journal articles in PMC produced by some publishers are prohibited to download and access automatically. We therefore complemented the publication list by submitting these SRA ID-PMID pairs to it manually.

Furthermore, in May 2011, NCBI began requiring that RNA sequencing (RNA-Seq), ChIP-sequencing (ChIP-Seq), and epigenomic data be submitted to the GEO [15], so for some transcriptomics data, only metadata is captured in SRA, while the raw sequence data are archived in GEO with the experimental designs stored as metadata [3]. Therefore, in addition to collecting SRA ID-PMID pairs, we retrieved the GEO IDs and related PMIDs and converted the GEO IDs to the corresponding SRA IDs. Accordingly, we retrieved 2748 SRA ID-PMID pairs as of October 2012.

### Characterization of SRA entries with disease keywords

To characterize the SRA entries by diseases, we utilized MeSH controlled vocabulary [7]. MeSH was originally designed for the indexing of MEDLINE articles by the NLM, thus we constructed SRA ID-MeSH pairs by extracting assigned MeSH terms from retrieved journal articles and restricted these pairs to disease related one. Accordingly, we obtained 989 SRA ID-disease MeSH pairs. A list of associations between SRA entries and related diseases, showing the SRA title, related disease, and hyperlinks to source articles is provided by our web service. We also constructed a disease frequency list (Table 1), according to the number of associated SRA entries.

**Table 1:** List of top 10 diseases extracted from the Sequence Read Archive (SRA).

Disease name	Online Mendelian Inheritance in Man (OMIM) ID	Number of projects
Breast Neoplasms	114480	43
Prostatic Neoplasms	176807	22
Disease Models, Animal	N/A	21
Genetic Predisposition to Disease	N/A	20
Disease Progression	N/A	15
Translocation, Genetic	N/A	14
Cell Transformation, Neoplastic	N/A	12
Lung Neoplasms	N/A (211980)	11
Staphylococcal infections	N/A	10
Malaria	N/A (611162)	9

We extracted disease terms of the Medical Subject Headings (MeSH) from assigned journal articles referring to the SRA entries. The MeSH disease category contains not only the disease name but also symptoms. The OMIM ID was converted from the MeSH terms to the Disease Name by using the Disease Ontology (DO). “Lung Neoplasms” should be assigned to the OMIM entry “Lung Cancer” (OMIM ID: 211980); however, there is no link in the DO.

We previously used assigned relevant MeSH terms to establish feature profiles OMIM entries on another web service called Gendoo. For example, Gendoo shows that Alzheimer Disease (OMIM ID: 104300) is related to the MeSH terms "Alzheimer Disease" for disease, "Amyloid Beta Protein" for chemicals and drugs, and "Brain" for anatomy. Moreover, Gendoo can illustrate the differences between diseases: type 1 diabetes mellitus (OMIM ID: 222100) is associated with "Autoimmune Disease" and "Spleen", and type 2 diabetes mellitus (OMIM ID: 125853) is associated with "Obesity" and "Adipocytes" [9], [10]. In the present study, we made connections between target diseases extracted from SRA entries and feature profiles in Gendoo by using DO. We established links between 56 diseases and 303 keywords extracted from SRA to corresponding Gendoo entries.

A keyword search involves the problem of lexical variations, such as "breast cancer" and "breast neoplasms". Therefore, we provide a tree view of diseases, and researchers can narrow their search by disease category using this list.

## Discussion

### Trends and growth of SRA entries

**Figure 2:**
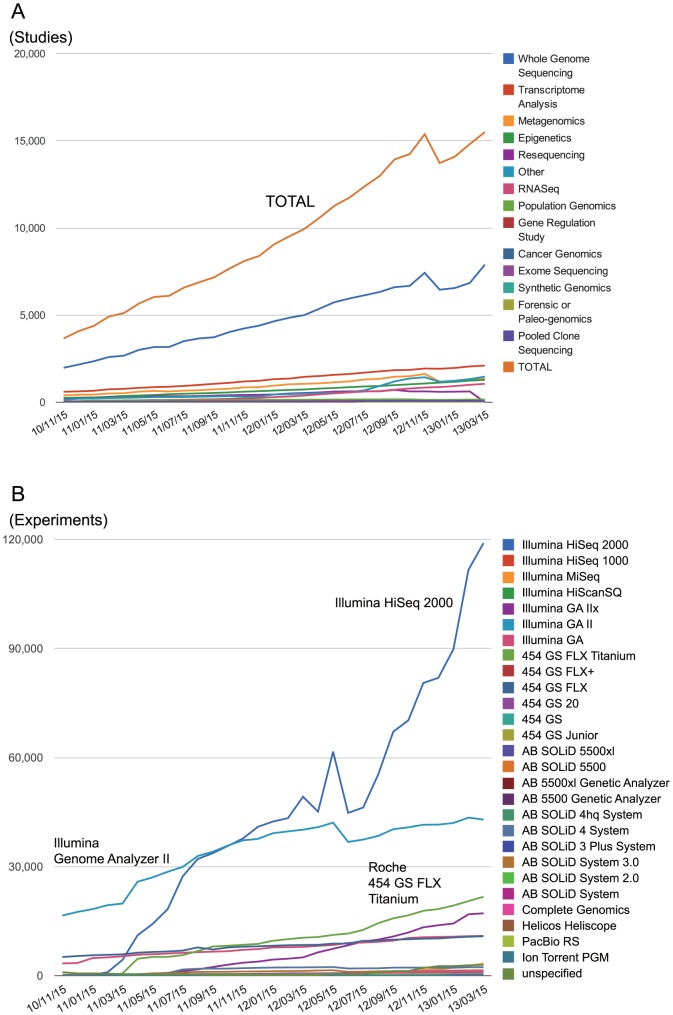
The growth of SRA data categorized by project types, and sequencing platforms. (A) The growth of the number of SRA studies categorized by project types. The number of studies are double that of the previous year. (B) The growth of the number of SRA experiments categorized by sequencing platforms. Over 200,000 experiments are submitted under approximately 14,000 studies. The experiments using Illumina HiSeq 2000 are dramatically increasing.

The high-throughput sequencing technology is utilized in various purposes including whole genome sequencing, transcriptome analysis, and metagenomics. We extracted such study type information from each study file and classified SRA projects according to it (Figure 2A). SRA contains approximately 14,000 projects as “study”, which is double that of the previous year. About a half of projects is an entry for whole genome sequencing (6630 projects). The major study types of other half are transcriptome analysis, metagenomics, and epigenetics (1983, 1240, and 1183 projects, respectively).

Further, we organized SRA dataset by sequencing platform (Figure 2B). Each study consists of multiple experiments: SRA archives over 200,000 experiments under 14,000 projects. Before 2010, Illumina Genome Analyzer II was the most popular sequencer, but in 2012, experiments using Illumina HiSeq 2000 were drastically increased. The top 3 of most used sequencer are Illumina HiSeq 2000, Illumina Genome Analyzer II, and 454 GS FLX Titanium (92,888, 42,274, and 19,463 experiments, respectively), and experiments with new sequencing platforms such as Complete Genomics, Helicos HeliScope, PacBio RS, and Ion Torrent PGM are also archived (1133, 437, 431, and 304 experiments, respectively).

Additionally, we categorized SRA data by species of samples. Table 2 shows the top-15 list of sample species in SRA studies. According to NCBI Taxonomy ID, 9060 taxonomic entries are referred in SRA database. Model organisms such as human, mouse, and fruit fly are major (1488, 898, and 322 projects, respectively) for approximately 14,000 projects, and many metagenome projects related to marine metagenome, soil metagenome are also performed (165 and 144 projects, respectively).

**Table 2:** List of top 15 species archived in SRA database.

Species of sample	The number of studies
*Homo sapiens*	1488
*Mus musculus*	898
unidentified	883
*Drosophila melanogaster*	322
*Caenorhabditis elegans*	206
*Arabidopsis thaliana*	191
marine metagenome	165
soil metagenome	144
*Saccharomyces cerevisiae*	142
*Escherichia coli* str. K-12 substr. MG1655	139
Bacteria	79
*Zea mays*	63
uncultured bacterium	60
*Danio rerio*	60
*Plasmodium falciparum*	55
Total	17319

We categorized SRA studies by species of samples. The number of total studies is larger than true one (*i.e.* approximately 14,000 studies) because one study can refer to multiple species. Model organisms such as human, mouse, and fruit fly are employed widely, and metagenome project are also intensively investigated.

We developed a project list that shows project titles and hyperlinks to corresponding experiment data, and researchers can restrict search results by study types, sequencers and species.

### SRA and other databases

In May 2011, NCBI announced it would handle RNA-Seq, ChIP-Seq and epigenomic data submitted to GEO, genomic and transcriptomic assemblies submitted to GenBank, and 16S ribosomal RNA with metagenomics submitted to GenBank [3]. After this announcement, many transcriptomic sequence data were archived not in SRA but in GEO. Therefore, we downloaded and parsed GEO data. Furthermore, recent projects are sometimes so large that the results are submitted to various databases, such as GenBank, GEO and SRA. Thus, the INSDC created a new database for a project, called “BioProject” [16], that made cross-references to various entries from the same projects. INSDC also launched a database for biological samples, called “BioSample” [16], because various experiments refer to the same samples. INSDC fosters the development of detailed data scheme of BioProject and BioSample, thus we plan to extend our web service to cover these databases and improve accessibility to archived data.

To retrieve easily public high-throughput sequencing data of sufficient quality for analysis, we collected articles referred to the IDs of GEO entry performed by massively parallel sequencers in conjunction with IDs of SRA. The resultant publication list is also useful to develop and evaluate the performance of software for assemble and mapping of high-throughput sequencing data. Researchers can restrict this list by study types and sequencers. Although about a half project of SRA entries is for whole genome sequencing (6630 projects) and transcriptome analysis is less than 15% (1983 projects) of 14,244 total projects, transcriptome analysis and epigenetics is referred by more journal articles (937 and 629 SRA ID-PMID pairs, respectively) than whole genome sequencing (599 pairs) for approximately 3000 of SRA ID-PMID pairs.

### Disease-relevant SRA entries

One of the major themes of molecular biology and medical science is to identify disease-relevant genes and to elucidate the mechanisms of action of the gene products. In SRA, a large amount of sequencing data is relevant to diseases and that archived as transcriptome analyses, epigenetics and cancer genome data.

Table S1 lists the top 10 projects captured in SRA database, determined by the number of experiments corresponding to each study. Four of the top 10 projects are related to disease, including the ARRA Autism Sequencing Collaboration (SRP003279), the Jackson Heart Study Allelic Spectrum Sequencing Discovery (SRP014601), and the Lung Adenocarcinoma Tumor Exome Sequencing Project (SRP012692) (the numbers of associated experiment files are 2257, 2030 and 1865, respectively). The Cancer Genome Atlas (TCGA) [17]–[21], however, included more than 30,000 experiments. The data were initially archived in SRA but were eventually moved to a website specific to the project.

Table 1 lists the top 10 terms of 303 disease keywords extracted from SRA entries. The list includes not only diseases but also symptoms. Restricting the list to diseases only, the major terms are “breast neoplasms”, “prostatic neoplasms” and “lung neoplasms” (43, 22 and 11 projects, respectively). The tree view of disease keywords also indicates that “neoplasms” is the area with the greatest focus. The SRA entries assigned to digestive system diseases and male urogenital diseases are also major because colorectal neoplasms and prostatic neoplasms, respectively, are included in these categories in addition to neoplasms sub-branch.

In this study, we constructed a disease-relevant project list that is referred by journal articles because we focused on entries referred by journal articles as quality evidence. We will increase disease-relevant entries by applying text-mining technology to descriptions and abstract sections of SRA database.

In addition, we made connections between disease extracted from SRA and corresponding feature profiles in Gendoo. Gendoo uses OMIM entries as a disease dictionary. OMIM contains disease-relevant genes and genetic disorders; subtypes of genetic diseases are also captured in OMIM. For example, “Diabetes Mellitus, Noninsulin-dependent, 3” (OMIM ID: 6036894) is a genetic subtype of type 2 diabetes mellitus (OMIM ID: 125853), which is linked to chromosome 20q12-q13.1. The differences between clinical features of NIDDM genetic subtypes are unclear, although the genetic mechanisms are probably different. In this study, we used DO to map diseases extracted from SRA to corresponding Gendoo entries because DO integrates and connects various ontologies, including MeSH, OMIM and ICD9CM [22]. In this case, the MeSH term “Lung Neoplasms” is not mapped to the OMIM entry “Lung Cancer” (OMIM ID: 211980). Inclusion or exclusion of these genetic subtypes in ontology is a matter of policy. Here, we plan to improve on the connections between MeSH and OMIM to link SRA disease entries to the Gendoo feature profiles by using MeSH terms corresponding to the feature profiles in Gendoo without genetic subtypes. Additionally, in OMIM, although entries for both type 1 and type 2 diabetes mellitus are present, an entry for simply “diabetes mellitus” is missing. We plan to use other dictionaries and ontologies of diseases, such as DO, to complete these missing entries.

## Conclusions

We characterized public high-throughput sequencing data by experimental designs including study type, sequencer, and species described in metadata. In addition, we produced a publication list to search SRA entries with enough quality to analysis, and a disease list by extracting disease MeSH terms from retrieved journal articles. We developed a web-based service called DBCLS SRA to visualize these lists. DBCLS SRA will accelerate to find and analyze datasets of interests.

## Supporting Information

Figure S1 **Data Structure of SRA.** The Sequence Read Archive (SRA) provides experiment designs along with raw sequences as metadata. The metadata composes of six objects of XML files: submission, study, experiment, run, sample, and analysis. The relationships between objects can be one-to-many. Original content is on http://trace.ddbj.nig.ac.jp/dra/metadata_e.html.
(EPS)

Figure S2 **A schematic view of pipeline for generating SRA-PMID pairs.** First, PubMed IDs (PMIDs) cited in reference section of the Sequence Read Archive (SRA) database are collected (a). Next, SRA IDs referred in abstracts of MEDLINE articles are extracted (b). In addition, SRA IDs described in the full-text version of articles in PubMed Central (PMC) and the website of the journals are parsed. Some transcriptome data using NGS are submitted not only to SRA but also to Gene Expression Omnibus (GEO), and reference articles often cite GEO IDs as links to the archived data. We therefore PMIDs cited in GEO database (c), and GEO IDs referred in MEDLINE, PMC, and journal websites (d).
(EPS)

Table S1 List of top 10 projects in the Sequence Read Archive (SRA). We sorted the projects archived in SRA database according to the number of assigned experiment files. Four of the top 10 projects were related to diseases (shown by asterisk *). The Cancer Genome Atlas (TCGA), containing over 30,000 experiments, was initially archived in SRA but eventually was moved to a website specific to that project.
(DOC)
