# Supplementary material for: Experimental Design-Based Functional Mining and Characterization of High-Throughput Sequencing Data in the Sequence Read Archive
Source: PLoS One. 2013 Oct 22;8(10):e77910. doi: 10.1371/journal.pone.0077910 (PMC3805581; doi:10.1371/journal.pone.0077910)
Supplement: Table S1 — List of top 10 projects in the Sequence Read Archive (SRA). We sorted the projects archived in SRA database according to the number of assigned experiment files. Four of the top 10 projects were related to diseases (shown by asterisk *). The Cancer Genome Atlas (TCGA), containing over 30,000 experiments, was initially archived in SRA but eventually was moved to a website specific to that project. (DOC) [file pone.0077910.s003.doc]

Table S1

| **SRA Study ID** |  | **Project title** | **Number of experiments** |
| --- | --- | --- | --- |
| ERP000426 |  | ZF_MrSol | 2718 |
| SRP011970 |  | Genetic Dissection of Model Complex Trait Using the Drosophila Synthetic Population Resource | 2640 |
| ERP000190 |  | Plasmodium_falciparum_natural_genome_variation | 2455 |
| SRP002163 |  | Human Microbiome Project Metagenomes Production Phase | 2404 |
| SRP003279 | * | ARRA Autism Sequencing Collaboration | 2257 |
| SRP014601 | * | Jackson Heart Study Allelic Spectrum Sequencing Discovery | 2030 |
| SRP012692 | * | Lung Adenocarcinoma Tumor Exome Sequencing Project | 1865 |
| ERP001040 |  | High_resolution_QTL_mapping_in_an_outbred_population_of_mice_using_low_coverage_next_generation_sequencing | 1767 |
| SRP003341 |  | NHLBI GO-ESP: Women's Health Initiative Exome Sequencing Project (WHI) | 1741 |
| SRP011021 | * | Prostate Cancer Genome Sequencing Project | 1640 |
